# Partial stratified ranked set sampling scheme for estimation of population mean and median

**DOI:** 10.1371/journal.pone.0275340

**Published:** 2023-02-15

**Authors:** Maria M, Ibrahim M. Almanjahie, Muhammad Ismail, Ammara Nawaz Cheema

**Affiliations:** 1 Department of Statistics, COMSATS University, Islamabad, Lahore, Pakistan; 2 Department of Mathematics, College of Science, King Khalid University, Abha, Saudi Arabia; 3 Statistical Research and Studies Support Unit, King Khalid University, Abha, Saudi Arabia; 4 Department of Mathematics, Air University, Islamabad, Pakistan; Universita degli Studi di Genova, ITALY

## Abstract

Ranked set sampling is an alternative to simple random sampling, which uses the least amount of money and time. The ranked set sampling (RSS) is modified to obtain a more efficient and cost-effective estimator of population parameters. This paper aims to bring a more efficient and cost-effective design than stratified ranked set sampling and simple random sampling. In some distributions, the suggested method used fewer sample units than stratified ranked set sampling and gives a more efficient estimation of population parameters. In symmetric distributions, the proposed design, called "partial stratified ranked set sampling" yields an unbiased estimator of the population mean. The design is illustrated with practical data of COVID-19 confirmed cases.

## 1. Introduction

Obtaining a more efficient and cost-effective population mean estimation is one of the researcher’s major goals. This goal can be accomplished by modifying the selection procedure of existing sampling designs. RSS design provides a more efficient, economical, and unbiased estimation of the population parameter. RSS used a cost-free ranking mechanism of the sampling or experimental units, which reduces the cost of a survey. Mclntyre [1] was the first who proposed RSS as a sampling design for population mean estimation. Takahasi and Wakimoto [2] investigated that RSS provides an unbiased estimation of the population mean. They also verified that the sample mean under RSS is more precise than sample mean of simple random sampling (SRS). Dell and Clutter [3] showed that, regardless of whether the ranking is flawless or not, the sample mean based on RSS provides unbiased estimation of the population mean and is at least as successful as the sample mean based on SRS. Stokes [4] explained that concomitant variables that are easily available could be used for the ranking of the variable of interest. Al-Omari et al. [5] introduced simple and generalized Z ranked set sampling schemes (ZRSS). They demonstrated that population mean estimators based on the proposed designs produce more efficient results for non-uniform distributions. Chen et al. [6] studied the maximum likelihood estimator (MLE) of the location parameter based on the moving extremes ranked set sampling (MERSS). For more modified schemes of RSS, see Al-Nasser et al. [7], Bani-Mustafa et al. [8], Samawi [9], Salehi and Ahmadi [10], Majd and Saba [11], Sevinc et al. [12], Khan et al. [13] and Ali et al. [14], Monjed et al. [15]. For different efficient classes of estimators under RSS and stratified ranked set sampling (StRSS), see Bhushan et al. [16–21].

Samawi [22] extended the ordinary RSS design to the StRSS scheme. He suggested that samples under RSS to be selected from each stratum. He conducted an empirical investigation and found that StRSS was a better predictor of the population mean than traditional stratified simple random sampling (StSRS). Samawai and Saeid [23] presented the stratified extreme ranked set sampling (StERSS) design to estimate the population mean. In their suggested scheme, the population was divided into ’H’ strata, then extreme ranked set samples were identified from each stratum. Ibrahim et al. [24] presented the stratified median ranked set sampling (StMRSS) design. In their scheme, the MRSS was used for the selection of samples from each stratum. They showed by simulations that StMRSS was more efficient than some of its counterpart designs. Al-Omari et al. [25] investigated the stratified percentile ranked set sampling (StPRSS) method. They conducted a numerical study and showed that StPRSS based mean estimator was more efficient than mean based on some of its counterpart designs. Mahdizadeh and Zamanzade [26] proposed the Stratified Pair Ranked Set Sampling (StPRSS) scheme. They showed that the suggested design provided a more effective estimation of the mean and utilized minimum cost compared to the StRSS method. Stratified unified ranked set sampling (StURSS) with flawless ranking was proposed by Chainarong et al. [27]. In the presence of outliers, Ali et al. [28] proposed stratified extreme-cum-median ranked set sampling (StEMRSS) to estimate the mean of heterogeneous populations. They demonstrated that, when compared to other StRSS systems, StEMRSS works well. Under lacking observations, Viada and Allende [29] created StRSS. The information for estimating the mean is completed using imputation based on ratio principles. They covered the necessary aspects of imputation and selection of sample processes. They used RSS models to compute imputation for stratified populations. In RSS, no actual measurements are made; instead, the units within each sample are sorted visually. When the data comes in batches of different sizes, the ranking is difficult and produces big inaccuracies, or it takes a long time. In this paper, a partial stratified ranked set sampling (PStRSS) design is proposed, a very effective design when all the experiment units are not available at same time. The suggested design selects the units using StRSS and SRS methods, i.e., ’c’ units are selected using StRSS and ‘d’ units using SRS. Thus, the total sample size n = c+d is selected. As a result, it is more effective than SRS and needs less sampling units and rankings than the RSS. Section 2 provides some existing ranked set sampling designs. In section 3, we present our proposed design and compare it with other designs through simulation studies. Section 4 illustrates the proposed design using real data, while inferences and final remarks are presented in section 5.

## 2. Some existing ranked set sampling designs

### 2.1 Ranked set sampling

The following is how a size *n* ranked set sample is chosen: Choose *n*^2^ elements randomly from a target population and divide them into *n* sets of *n* varying sizes. The selected units within each group are then ranked visually or using any other low-cost technique. From the first set the least ranked unit is identified. The second least ranked unit from the second set is identified. The process is continued until the highest-ranking unit from the last set is selected. This technique can be performed *r* times to obtain *rn* RSS units. Consider the research variable *X*, which has a probability density function *f*_*x*_(*x*), a cumulative density function *F*_*x*_(*x*), a mean *μ*_*x*_ and a variance $\sigma_{X}^{2}$. Let $X_{11j},X_{12j},...,X_{1nj},X_{21j},X_{22j},...,X_{2nj},X_{n1j},X_{n2j},...,X_{nnj}$ be the *n* independent simple random sample each of size *n* from *j*^*th*^ cycle taken from *f*_*x*_(*x*), where *j* = 1,2,…,*r*. The RSS mean and variance are as follows,

$${\bar{X}}_{RSS}=\frac{1}{nr}\sum_{j=1}^{r}\sum_{i=1}^{n}X_{i(i:n)j}$$ (1)

The variance is as follows,

$$\mathrm{var}\left({\bar{X}}_{RSS}\right)=\frac{\sigma_{X}^{2}}{rn}-\frac{1}{rn^{2}}{\sum_{i=1}^{n}\left(\mu_{i(i:n)}-\mu \right)}^{2}$$ (2)

where, *μ*_*i*(*i*:*n*)_ is the mean of *i*^*th*^ order statistics and $\sigma_{X}^{2}$ is the variance of SRS.

### 2.2 Stratified ranked set sampling

The StRSS procedure divides the population into H mutually exclusive and exhaustive strata in order to obtain a size *n* sample. Then, from the stratum *h* in *r*_*h*_ cycle, select an independently ranked set sample of size *j*_*h*_*n*_*h*_ units. Let $X_{(i:n)j}^{h}$ be the *i*^*th*^ judgment order statistic in the *j*^*th*^ cycle of the ranked set sample taken from stratum *h*. The observations $X_{(i:n)j}^{h}$ $\left(h=1,....,H;i=1,..,n_{h};j=1,...r_{h}\right)$ are independent, but not distributed identically. The common mean and variance for fixed *h* and *i*, $X_{(i:n)j}^{h}$‘s (*j* = 1,…*r*_*h*_) are distributed identically, and are denoted by *μ*_(*i*:*n*)*h*_ and *σ*^2^_(*i*:*n*)*h*_ respectively. The population mean under StRSS is given by,

$${\bar{X}}_{\left(StRSS\right)}=\sum_{h=1}^{H}\frac{N_{h}}{N}{\bar{X}}_{\left(RSS,h\right)},$$ (3)

where,

${\bar{X}}_{\left(StRSS,h\right)}=\frac{1}{n_{h}r_{h}}\sum_{i=1}^{n_{h}}\sum_{j=1}^{r_{h}}X_{[i:n]j}^{h}$ is the mean estimator of RSS in stratum *h*. The variance is as follows,

$$\mathrm{var}({\bar{X}}_{\left(StRSS\right)})=\sum_{h=1}^{H}{\left(\frac{N_{h}}{Nn_{h}}\right)}^{2}\frac{1}{r_{h}}\sum_{i=1}^{n_{h}}\sigma_{\left[i:n\right]}^{2}{}_{h},$$ (4)

where

$$\sum_{i=1}^{n_{h}}\sigma_{[i:n]h}^{2}=n_{h}\sigma_{h}^{2}-\sum_{i=1}^{n_{h}}{\left({\bar{X}}_{(i,n)h}-\mu_{h}\right)}^{2}.$$ (5)

Combining (4) and (5) we get,

$$\mathrm{var}({\bar{X}}_{StRSS})=\sum_{h=1}^{H}{\left(\frac{N_{h}}{Nn_{h}}\right)}^{2}\frac{1}{r_{h}}(n_{h}\sigma_{h}^{2}-\sum_{i=1}^{n_{h}}{\left({\bar{X}}_{\left(i,h\right)}-\mu_{h}\right)}^{2}),$$ (6)

$$=\sum_{h=1}^{H}{\left(\frac{N_{h}}{N}\right)}^{2}\frac{\sigma_{h}^{2}}{n_{h}r_{h}}-\sum_{h=1}^{H}{\left(\frac{N_{h}}{N}\right)}^{2}\frac{1}{n_{h}^{2}r_{h}}(\sum_{i=1}^{n_{h}}{\left({\bar{X}}_{(i,n_{h})h}-\mu_{h}\right)}^{2}),$$

$$\mathrm{var}({\bar{X}}_{\left(StRSS\right)})=\mathrm{var}({\bar{X}}_{\left(StSRS\right)})-\sum_{h=1}^{H}{\left(\frac{N_{h}}{N}\right)}^{2}\frac{1}{n_{h}^{2}r_{h}}\sum_{i=1}^{n_{h}}{\left({\bar{X}}_{(i,n_{h})h}-\mu_{h}\right)}^{2}.$$ (7)

## 3. Proposed design

In this section, PStRSS design is suggested. The design is effective for some distributions when the required units for the StRSS method are not available or arrived at batches. Then some units are selected by the StRSS method, and others are selected using the SRS method. The procedure of geting PStRSS with sample size n is as follows:

1. Define coefficient *c* such that *c* = *βn*, where 0≤*β*≤0.5.
2. Select 2*c* units from the population using simple random sampling technique.
3. The remaining *n*−2*c* units are selected as; divide the population into H mutually exclusive and exhaustive strata. Then, select independently ranked set sample of size *j*_*h*_*d*_*h*_ (for *d* = *n*−2*c*) units from stratum *h* in *j*^*th*^ cycle.
4. The above procedure can be repeated *r* time in order to get *rn* units.

### 3.1 Estimation

The population mean estimator under PStRSS is as below,

$${\bar{X}}_{\left(PStRSS\right)}=\sum_{h=1}^{H}\frac{N_{h}}{N}{\bar{X}}_{\left(StRSS,h\right)},$$

where,

$${\bar{X}}_{\left(PStRSS,h\right)}=\frac{1}{n_{h}r_{h}}\sum_{h=1}^{H}\frac{N_{h}}{N}\sum_{j=1}^{rh}\left(\sum_{i=1}^{c}X^{h}{}_{i}+\sum_{i=c+1}^{n-c}X_{(i:n)j}^{h}+\sum_{i=n-c+1}^{n}X^{h}{}_{i}\right),$$ (8)

Variance of ${\bar{X}}_{\left(PStRSS,h\right)}$ is as under,

$$\mathrm{var}({\bar{X}}_{\left(PStRSS,h\right)})=\frac{1}{n_{h}{}^{2}r_{h}{}^{2}}\sum_{h=1}^{H}\frac{N^{2}{}_{h}}{N^{2}}\sum_{j=1}^{rh}\left(2c\sigma_{X}^{2}+\sum_{i=c+1}^{n-c}\mathrm{var}(X_{(i:n)j}^{h})\right).$$ (9)

### Lemma 1

${\bar{X}}_{\left(PStRSS,h\right)}$ is an unbiased estimator of *μ* for symmetric distributions.

#### Proof

Taking expectation on both sides of Eq (8) we have,

$$E({\bar{X}}_{\left(PStRSS,h\right)})=\frac{1}{n_{h}r_{h}}\sum_{h=1}^{H}\frac{N_{h}}{N}\sum_{j=1}^{rh}\left(\sum_{i=1}^{c}E(X^{h}{}_{i})+\sum_{i=c+1}^{n-c}E(X_{(i:n)j}^{h})+\sum_{i=n-c+1}^{n}E(X^{h}{}_{i})\right),$$ (10)

$$E({\bar{X}}_{\left(PStRSS,h\right)})=\frac{1}{n_{h}r_{h}}\sum_{h=1}^{H}\frac{N_{h}}{N}\sum_{j=1}^{rh}\left(\sum_{i=1}^{c}\mu_{h}+\sum_{i=c+1}^{n-c}\mu_{\left(i:n,h\right)}+\sum_{i=n-c+1}^{n}\mu_{h}\right),$$

$$E({\bar{X}}_{\left(PStRSS,h\right)})=\frac{1}{n_{h}r_{h}}\sum_{h=1}^{H}\frac{N_{h}}{N}\sum_{j=1}^{rh}\left(2c\mu_{h}+(n_{h}-2c)\mu_{\left(i:n,h\right)}\right),$$

$$E({\bar{X}}_{\left(PStRSS,h\right)})=\frac{1}{n_{h}r_{h}}\sum_{h=1}^{H}\frac{N_{h}}{N}\sum_{j=1}^{rh}\left(2c\mu_{h}+n_{h}\mu_{\left(i:n,h\right)}-2c\mu_{\left(i:n,h\right)}\right),$$ (11)

In symmetric distribution *μ*_(*i*:*n*,*h*)_ = *μ* (David and Nagaraja, [30])

$$E({\bar{X}}_{\left(PStRSS,h\right)})=\frac{1}{n_{h}r_{h}}\sum_{h=1}^{H}\frac{N_{h}}{N}\sum_{j=1}^{rh}\left(2c\mu +n_{h}\mu_{h}-2c\mu \right),$$

$$E({\bar{X}}_{\left(PStRSS,h\right)})=\frac{1}{n_{h}r_{h}}\sum_{h=1}^{H}\frac{N_{h}}{N}\sum_{j=1}^{rh}n_{h}\mu_{h},$$

$$E({\bar{X}}_{\left(PStRSS,h\right)})=\sum_{h=1}^{H}W_{u}\mu_{h},$$

$$E({\bar{X}}_{\left(PStRSS,h\right)})=\mu .$$

### 3.2 Simulation

The mean estimator’s performance under PStRSS is compared with the mean estimator of StRSS by conducting a simulation study. The efficiency of the PStRSS based mean estimator is evaluated using both symmetric and non-symmetric distributions. The distributions consider for simulation study are, Normal (0,1), Lognormal (0,1), Weibull (0.5,1) and Logistic (0,1). Relative efficiency (REs) of PStRSS and StRSS concerning SRS is investigated using 50,000 iterations from two strata. The simulation is done by R 4.5 software. The sample is taken from two strata: (4,4), (5,5) and (6,8).

The equation used for REs of symmetric distributions is as below,

$$RE({\bar{X}}_{t},{\bar{X}}_{SRS})=\frac{\mathrm{var}({\bar{X}}_{SSRS})}{\mathrm{var}({\bar{X}}_{t})}$$

where, *t* defines the sampling methods as, PStRSS and StRSS.

The equation used for REs of asymmetric distributions is as below,

$$RE({\bar{X}}_{t},{\bar{X}}_{SRS})=\frac{\mathrm{var}({\bar{X}}_{SSRS})}{MSE({\bar{X}}_{t})}$$

where, *t* defines the sampling method as, PStRSS.

**Fig 1:**
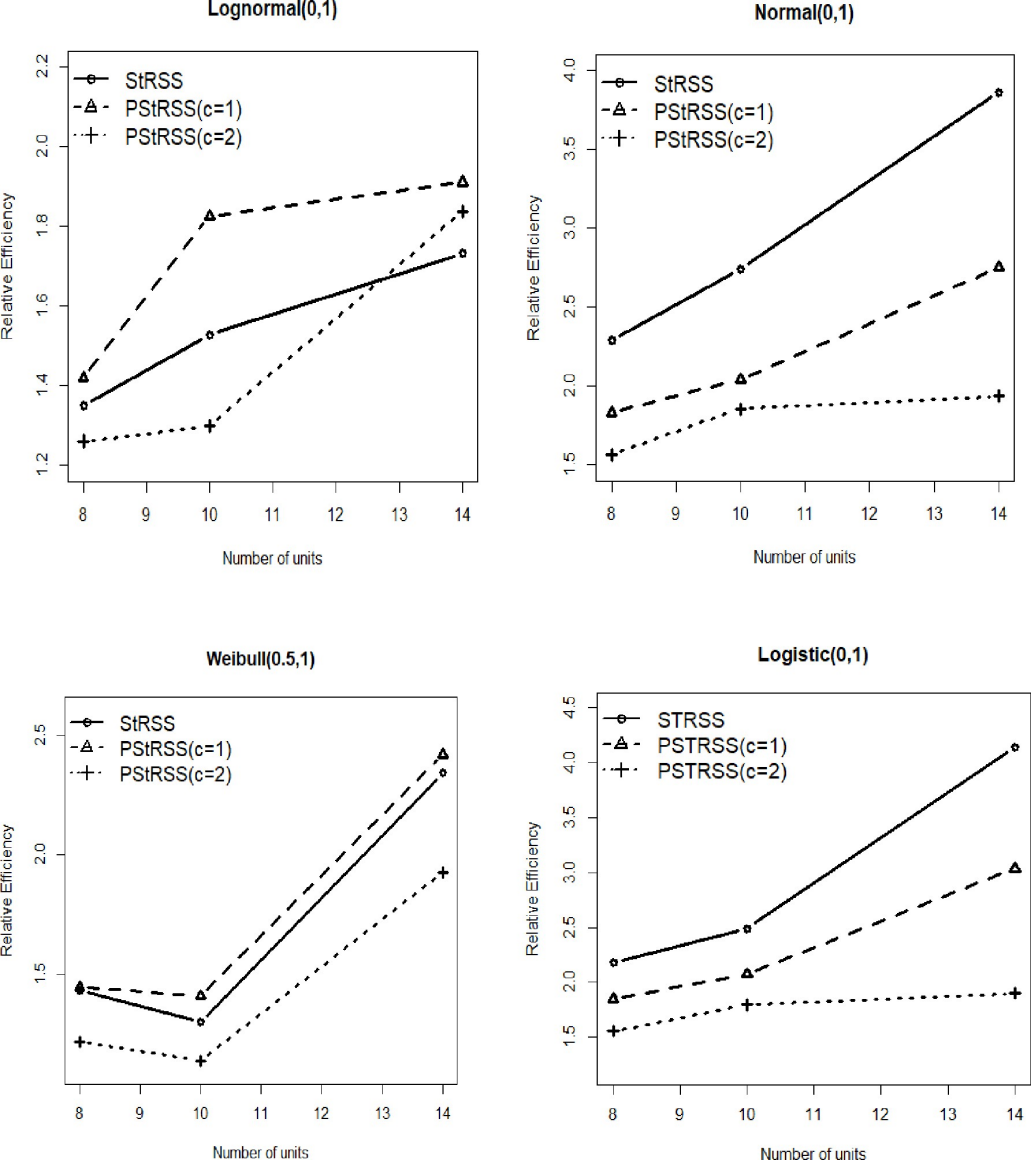
Relative efficiency of mean estimator based on PStRSS, StRSS relative to SRS.

In Fig 1 the REs is presented. The Fig 1 demonstrates that for Normal (0,1) and Logistic (0,1) distributions, the StRSS have higher REs than PStRSS (c = 1) and PStRSS (c = 2). But StRSS utilized more units than PStRSS. Moreover, the REs of the mean estimator of PStRSS for given distributions is higher than SRS because the REs is higher than one. Fig 1 further shows that for Weibull (0.5,1) and Lognormal (0,1), the REs of the mean estimator of suggested design PStRSS (c = 1) is higher than StRSS and SRS. But for the mean estimator of PStRSS (c = 2), the REs is higher than StRSS in a high sample size. In Lognormal (0,1), PStRSS (c = 1) outperforms StRSS in terms of mean estimator performance. While for PStRSS (c = 2), the REs is lower than StRSS. Thus, Fig 1 shows that PStRSS for c = 1,2 is more efficient than SRS. Simultaneously, the suggested design is more efficient in some distributions depending on the value of ‘c’ and sample size.

### 3.3 Estimation of the median

For the skewed type of distributions, the median as a measure of location is recommended. For example, the distributions of income, production, and expenditure are skewed. In this section, the median estimator under the suggested scheme PStRSS is investigated. The performance of PStRSS’s median estimator is tested in a simulation study. For this purpose, R 4.5 software is used, and the simulation is repeated 50,000 times to estimate the mean square error of the median estimator under PStRSS for c = 1 and 2.

The REs for the estimator of median for *h*^*th*^ stratum is given as,

$$RE\left({\bar{X}}_{t},{\bar{X}}_{SRS}\right)=\frac{MSE({\hat{\theta }}_{SRS})}{MSE({\hat{\theta }}_{v})},$$

where, *t* defines the sampling methods as, PStRSS, StRSS.

**Fig 2:**
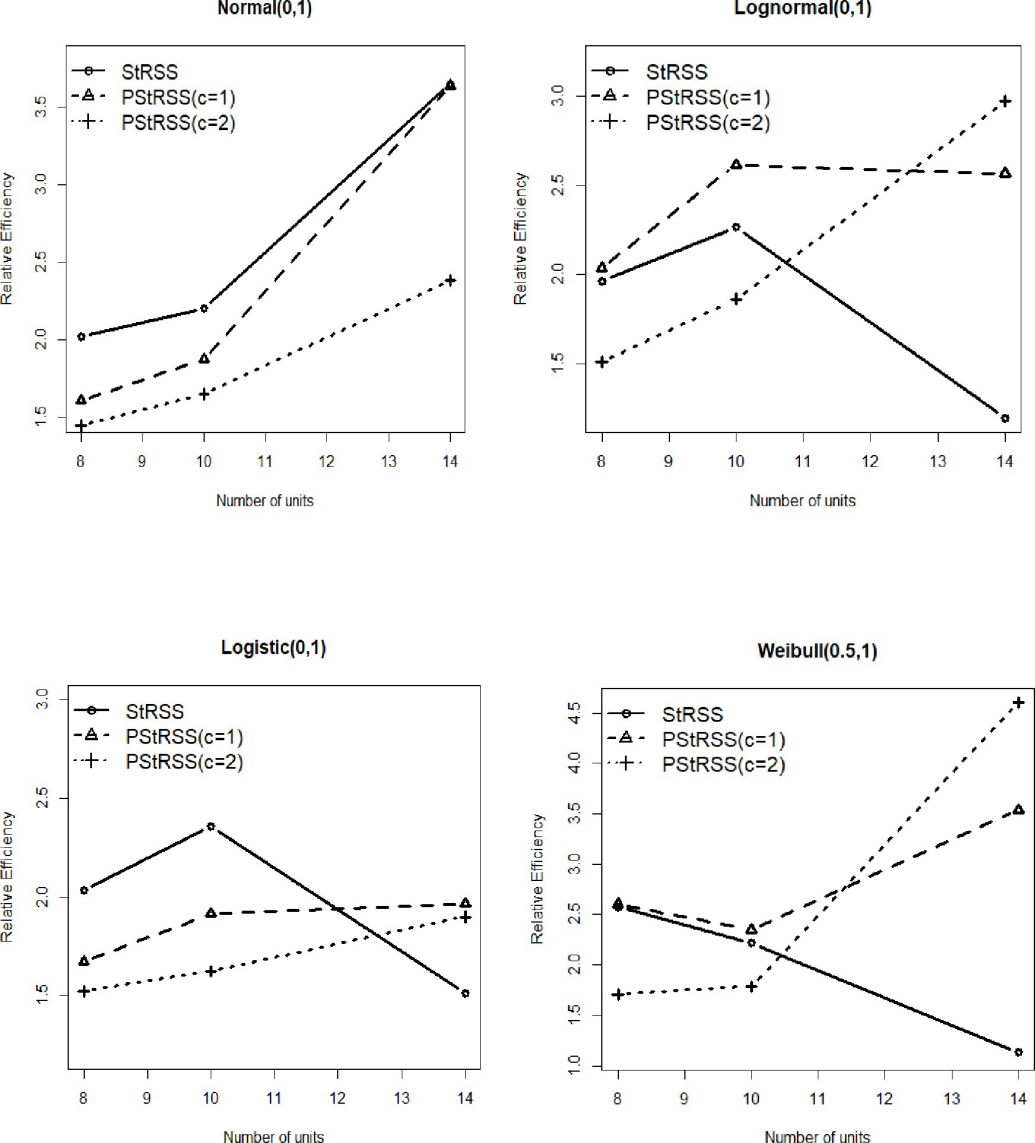
Relative efficiency of mdian estimator based on PStRSS, StRSS relative to SRS.

The result of the simulation study is presented in Fig 2. For Normal (0,1) distribution, the StRSS is performing better than PStRSS for c = 1 and 2. While in Lognormal (0,1) distribution, the suggested scheme PStRSS (c = 1) performs better than StRSS in estimating the median. In PStRSS (c = 2), the median’s estimation is precise than StRSS for a large sample size. Fig 2 further reveals that the median estimator under PStRSS for c = 1,2 estimates the median more efficiently than StRSS for large sample size. For Weibull (0.5,1), the efficiency of estimating the median estimator under PStRSS (c = 1) is higher than StRSS, while for PStRSS (c = 2), the efficiency is higher for a large sample only. The Fig 2 suggests that the PStRSS for c = 1 and c = 2 estimate the population median more efficiently than the SRS based median estimator.

## 4. An application to COVID-19 confirmed cases in Pakistan

The Coronavirus, 2019 is a newly identified virus that causes an infectious disease. The Coronavirus (COVID-19 Cases Data, 2020, [31]) COVID-19 daily confirmed cases for the last two months, i.e., October and November 2020, in Pakistan is considered.

We are interested in selecting n = 4 samples (days) using the suggested scheme PStRSS, StRSS, and StSRS. Procedure of this scheme is applied to identify 4 samples. Months are considered as strata. The equal allocation method is used in PStRSS, StRSS, and StSRS designs. In PStRSS, 2 samples are selected by the StRSS method, and 2 samples are selected by the SRS method.

The mean and variance for the suggested scheme PStRSS, StRSS, and StSRS are presented in Table 1. Here n = 4 is the sample size and r = 1 is number of cycle. In the suggested scheme the PRSS samples are drawn from each stratum. For selection of a ranked set sample of size n = 4, the researcher must observe 16 units but due to limited budget and time, it is hard to apply the RSS technique. In this condition under the PStRSS the researcher need to observe only (4, 1) = 10 units, (4, 2) = 4 units from two strata. It is clear from Table 1 that the estimator of mean based on PStRSS not only outperforms as compared with their competitors based on StRSS and StSRS but also need less number of units than StRSS. The mean from population data in the PStRSS is 1219.333, which is closer to the population mean than StRSS and StSRS mean. Thus, the suggested scheme estimated the confirmed cases of COVID-19 in Pakistan more efficiently.

**Table 1:** Summary statistics of population and samples taken at different sampling schemes for n = 4, r = 1.

Estimator	Population	PStRSS	StRSS	StSRS
Mean	1159.272	1219.333	948.630	1348.375
Variance	695978.5	473238.1	474177.4	474177.4

## 5. Conclusion and final remarks

This paper suggests a new efficient and cost-effective design, PStRSS. This design utilizes less sampling units than traditional StRSS and provides a more efficient mean and median estimator of the population in some distributions. Therefore, when the required sample sizes are not available to conduct StRSS, instead of switching to SRS, one should use PStRSS, which is extra efficient than SRS and, in some distributions, estimate the average and median more precisely. Our suggested design PStRSS outperforms for Normal (0,1) and Logistic (0,1) distributions than SRS and for Weibull (0.5,1) and Lognormal (0,1) it is more efficient than StRSS and SRS for PStRSS (c = 1). It is concluded that PStRSS for c = 1,2 is more efficient than SRS and outperforms StRSS in terms of mean estimator for c = 1. The suggested approach is also more effective in some distributions depending on the sample size and value of c. Moreover, the suggested scheme estimate the average confirmed cases of COVID-19 efficiently. It is recommended when daily confirmed cases of some area could not be found; then, the PStRSS sampling method should be used to estimate the daily confirmed cases of COVID-19.
